# Polyimide/Ionic Liquid Composite Membranes for Middle and High Temperature Fuel Cell Application: Water Sorption Behavior and Proton Conductivity

**DOI:** 10.3390/membranes10050082

**Published:** 2020-04-28

**Authors:** Kateryna Fatyeyeva, Sergiy Rogalsky, Stanislav Makhno, Oksana Tarasyuk, Jorge Arturo Soto Puente, Stéphane Marais

**Affiliations:** 1 Polymères Biopolymères Surfaces (PBS), Normandie University, UNIROUEN, INSA ROUEN, CNRS, 76000 Rouen, France; 2 Institute of Bioorganic Chemistry and Petrochemistry, National Academy of Science of Ukraine, 50, Kharkivske Schose, 02160 Kyiv, Ukraine; 3 Chuiko Institute of Surface Chemistry, National Academy of Sciences of Ukraine, 17, General Naumov St., 03164 Kyiv, Ukraine

**Keywords:** porous polyimide membrane, ionic liquid, conductivity, vapour sorption, thermal stability

## Abstract

Four water insoluble room-temperature protic ionic liquids (PILs) based on the *N*-alkylimidazolium cation with the alkyl chain length from 1 to 4 and bis(trifluoromethylsulfonyl)imide anion were synthesized and their chemical structure was confirmed by the ^1^H NMR and ^19^F NMR analysis. PILs were revealed to be thermally stable up to 360 and 400 °C. At the same time, the proton conductivity of PILs was found to be dependent mostly on the temperature and, to a less extent, on the type of the cation, i.e., the increase of the conductivity from ~3 × 10^−4^ S/cm at 25 °C to 2 × 10^−2^ S/cm at 150 °C was observed. The water vapour sorption capacity of PILs was evaluated as a function of relative humidity and the influence of the alkyl chain length on the phase behaviour in the PIL-water system was discussed. The composite polyimide/PILs membranes were prepared by the PIL immobilization in the porous polymer (Matrimid^®^ 5218) film. The composite membranes showed a high level of proton conductivity (~10^−3^ S/cm) at elevated temperatures (up to 160 °C). The obtained results reveal that the elaborated composite polyimide/PIL membranes are promising candidates for the application as proton exchange membrane at middle and high temperatures.

## 1. Introduction

The proton exchange membrane fuel cells (PEMFCs) are considered as one of the most promising sources of the green clean energy power alternative to the internal combustion engines and can be used in portable devices, transport, and small stationary power plants [1,2]. However, there are still many challenges until the time when this technology will be commercially viable. The main issues to be overcome are the high cost of fuel cell systems, high maintenance costs, and the short lifecycle [2]. The polymer electrolyte membrane (PEM), as a key component of PEMFC, has a crucial role as the transport medium for generating protons by the oxidation of fuel and, thus, determining the cost, compactness and the operational properties of a fuel cell in general [1,2,3]. Until recently, perfluoro-sulfonated polymers, such as Nafion^®^, Flemion^®^, or Hyflon^®^, remain the most commonly used PEMs [4,5]. However, their performance is strongly dependent on the hydration level and starts to become rather poor at above 90 °C and at low relative humidity, since water molecules act as the proton carriers and play a major role in the proton conduction of such polymer electrolytes [6]. For the last two decades, researchers tried to develop different hydrocarbon membranes for PEMFCs working at high temperatures [2,3,4,7]. Compared with Nafion^®^-based low temperature PEMFCs, the middle and high temperature PEMFCs (100 °C < T < 200 °C) have certain advantages such as the enhancement of electrode kinetics, easy water thermal management, and higher tolerance to the CO impurities present in the hydrogen fuel stream [3,6,7]. Thus, new stable electrolyte materials able to work in dry state at temperatures higher than 90 °C are required [7,8,9,10].

During the last few decades, ionic liquids (ILs) (i.e., organic salts composed of ions with the melting point below 100 °C [11]) attract extensive attention due to their outstanding properties, namely low volatility, high solvation capacity, large electrochemical window, high thermal stability, and conductivity. Due to a large choice of ions, various kinds of ILs can be obtained [10,11,12]. As the electrolyte used in fuel cells should be proton conductive, protic ILs (PILs) synthesized by the neutralization of Brønsted acid and base are often studied for PEMFCs. Usually, the conductivity of PILs ranges from 1 × 10^−4^ up to 1.8 × 10^−2^ S/cm at room temperature [12,13]. The protons are transported by the cations via the vehicle and Grotthuss mechanisms. The electrochemical reactions (i.e., hydrogen oxidation and oxygen reduction) can be further facilitated at the interface electrode/PILs [13]. However, PIL alone cannot form an electrolyte in PEMFC. Polymer membranes based on PILs may be prepared in three different ways: (1) the polymer membrane swelling with PIL [10,12,14,15], (2) dissolving the polymer and PIL in a common solvent and film casting [16,17,18], or (3) the polymerization of monomers in the PIL presence [12,19]. Besides, it is known that a good compatibility of the polymer and PIL is a necessary condition for the sufficient membrane conductivity [20]. For this study, the first approach, i.e., the swelling of a polymer film with PIL, has been chosen. In this case, the polymer serves as a matrix support possessing the sufficient mechanical strength, and the chemical and thermal stability. Numerous researches have been already performed to prepare the new composite PIL membranes with both high proton conductivity and mechanical stability based on different polymers as a mechanical support [10,14,21,22].

Among many advanced polymers, polyimides are considered as one of the serious candidates for high temperature PEMFCs due to their excellent thermomechanical properties, good film forming ability, chemical stability, and low methanol permeability [10,18,23,24,25]. Besides, polyimide-based membranes attract research attention due to possibility to work at high operation temperature of ~150 °C, thus allowing us to prevent the Pt poisoning. In most cases the sulfonated polyimide is used as a polymer matrix since it has much better solubility in amide organic solvents and contains sulfonic groups as additional proton carriers [23,25]. Moreover, the sulfonated polyimide is found to have better compatibility with PILs, thus preventing their desorption from the polymer matrix [24]. However, the presence of the sulfonic group on the polymer backbones may dramatically decrease their mechanical properties [23,25].

Usually, water soluble PILs (for example, imidazolium phosphate [10,18], diethylmethylammonium trifluoromethanesulfonate [25], triethylammonium trifluoromethanesulfonate [26], etc.) are used to obtain the proton conducting membranes based on various polyimide matrices. However, in order to realize the PIL-based membranes for fuel cell application, the hydrophobic PILs are necessary as the water formation at the cathode may result in the PILs leakage from the membrane during fuel cell operation [2]. So, the main idea of the present work is to use water insoluble PILs based on bis(trifluoromethylsulfonyl)imide anion. For this reason, four PILs were synthesized. As synthesized ionic liquids are intended to be used in fuel cells, they should possess high proton conductivity value. Therefore, the knowledge of the conductivity and ionic diffusion properties in PILs is essential. As the properties of PILs can be defined through the cation and anion selection [10,20,24], the influence of the alkyl chain length of the *N*-alkylimidazolium cation on the water vapour sorption properties, the proton conductivity and the thermal stability of PILs was analyzed. The choice of the imidazolium-based cation is based on its higher chemical stability and proton conductivity and lower viscosity in comparison with other PILs cations (such as pyridinium, pyrrolidinium, piperidinium, morpholinium, etc.) [14,17,21]. The performance of the composite polyimide/PIL membranes was evaluated in terms of their morphology, thermal, and mechanical stability. Furthermore, the electrochemical properties of the polyimide membranes incorporating PILs with the different alkyl chain length were compared.

## 2. Experimental

### 2.1. Materials

Imidazole (99.5%, Sigma-Aldrich, Saint-Quentin-Fallavier, France), sodium hydride (60% dispersion in mineral oil, Sigma-Aldrich), acetonitrile (Sigma-Aldrich), methyl iodide (99.0%, Sigma-Aldrich), bromoethane (98%, Sigma-Aldrich), 1-bromopropane (99%, Sigma-Aldrich), 1-bromobutane (99%, Sigma-Aldrich), hydrochloric acid (37%), lithium bis(trifluoromethylsulfonyl)imide (99%, Sigma-Aldrich), methylene chloride (Fluka), sodium sulphate (anhydrous) (99.0%, Sigma-Aldrich), 1-methyl-2-pyrrolidinone (NMP) (99.5%, Fluka, France), polyvinylpyrrolidone (PVP) (K 90, average M_w_ 360,000, Sigma-Aldrich) were used as received, except acetonitrile. Acetonitrile was purified according to [27].

Polyimide Matrimid^®^ 5218 powder was supplied from Huntsman society (Basel, Switzerland).

### 2.2. Synthesis of PILs

**Scheme 1:**
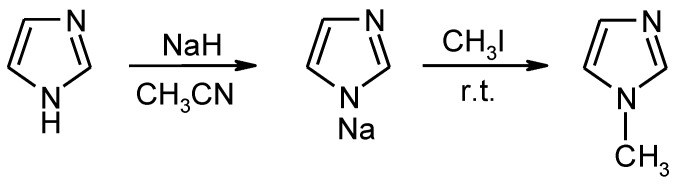
Synthesis of 1-methylimidazole.

1-methylimidazole was synthesized according to Scheme 1 [28]. For this purpose, sodium hydride (13 g, 60% suspension in mineral oil) was washed with hexane (60 mL), filtered off and added to 200 mL of dried acetonitrile. Imidazole (20 g, 0.29 mol) was added to the stirred suspension by portions and the reaction continued for 6 h. The mixture was cooled down to 0 to 5 °C and methyl iodide (43 g, 0.3 mol) was added dropwise. The reaction was carried out for 12 h at 25 °C. Acetonitrile was distilled off and the crude 1-methylimidazole was purified by the distillation with the help of the water-jet pump at 105 to 108 °C. 1-methylimidazole was converted into the hydrochloride salt by the dissolution in 100 mL of 10% hydrochloric acid. The solution was evaporated and the solid residue of 1-methylimidazolium chloride was dried at 100 °C.

**Scheme 2:**
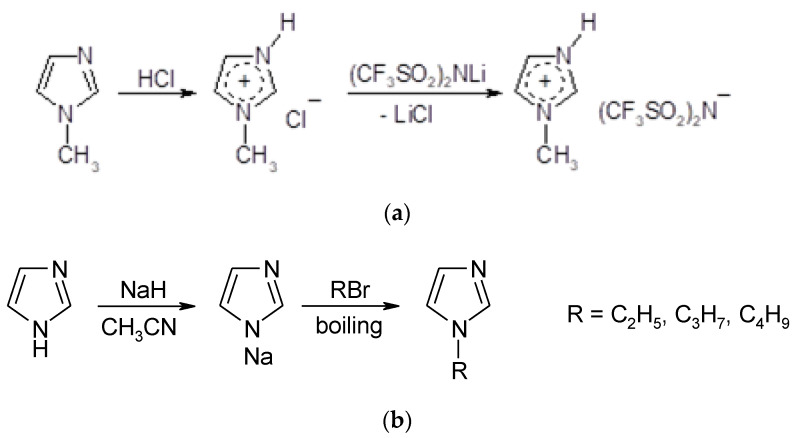
(**a**) Synthesis of 1-methylimidazolium bis(trifluoromethylsulfonyl)imide (MIM-TFSI); (**b**) synthesis of 1-alkylimidazole derivatives.

*1-methylimidazolium bis(trifluoromethylsulfonyl)imide* (MIM-TFSI) ionic liquid was prepared according to Scheme 2a. For this purpose, 12.1 g (0.042 mol) of lithium bis(trifluoromethylsulfonyl)imide were added to the stirred solution of 1-methylimidazolium chloride (5 g, 0.042 mol) in 50 mL of water. The formed amorphous solid product was filtered off and dissolved in 100 mL of ethyl acetate. The solution was washed with water (100 mL) and dried overnight with magnesium sulfate. Ethyl acetate was distilled off at the normal pressure. The residual solvent was removed under vacuum (1 mbar) at 70 °C for 12 h. Obtained PIL is the water insoluble solid of a light brown color with the melting point of 49 to 50 °C.

^1^H NMR (300 MHz, DMSO-*d_6_*): δ = 3.86 (s, 3H, CH_3_), 7.63 (m, 2H, C_4_-H, C_5_-H), 9.02 (s, 1H, C_2_-H). ^19^F NMR (188 MHz, DMSO-*d_6_*): δ = −75.2 (s, 6F).

1-ethylimidazole, 1-propylimidazole, and 1-butylimidazole were synthesized according to Scheme 2b. For this purpose, sodium hydride (13 g) was washed with hexane (60 mL), filtered off and added to 200 mL of dried acetonitrile. Imidazole (20 g, 0.29 mol) was added by portions to the stirred suspension and the stirring continued 6 h. Then, 0.3 mol of corresponding bromoalkane (bromoethane, 1-bromopropane, or 1-bromobutane) was added and the mixture was heated to boiling during 12 h. The formed precipitate of sodium bromide was removed by the filtration after cooling the mixture to room temperature. Acetonitrile was distilled off and 1-alkylimidazole was purified by the distillation with the help of the water-jet pump in the temperature range of 110 to 130 °C. 1-alkylimidazole was converted into the corresponding hydrochloride salt by the dissolution in 100 mL of 10% hydrochloric acid followed by the water evaporation. The solid residue of 1-alkylimidazolium chloride was dried at 100 °C.

**Scheme 3:**
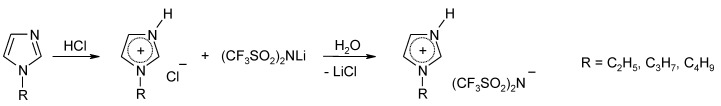
Synthesis of 1-alkylimidazolium-TFSI ionic liquids.

1-alkylimidazolium bis(trifluoromethylsulfonyl)imide ionic liquids were prepared according to Scheme 3. 12.1 g (0.042 mol) of lithium bis(trifluoromethylsulfonyl)imide were added to the stirred solution of corresponding 1-alkylimidazolium chloride (0.042 mol) in 50 mL of water. The formed bottom layer of PIL was extracted with methylene chloride (2 × 70 mL). The organic layer was washed with water (100 mL) and dried overnight with magnesium sulfate. Methylene chloride was distilled off at the normal pressure. The residual solvent was removed under vacuum (1 mbar) at 70 °C for 12 h. The obtained products are low viscous colourless liquids.

1-ethylimidazolium bis(trifluoromethylsulfonyl)imide (EIM-TFSI):

^1^H NMR (300 MHz, DMSO-*d_6_*): δ = 1.47 (m, 3H, CH_3_), 4.24 (m, 2H, CH_2_), 7.64-7.73 (m, 2H, C_4_–H, C_5_–H), 9.08 (s, 1H, C_2_–H), 10.72 (br s, 1H, NH)

^19^F NMR (188 MHz, DMSO-*d_6_*): δ = −79.9.

1-propylimidazolium bis(trifluoromethylsulfonyl)imide (PIM-TFSI):

^1^H NMR (300 MHz, DMSO-*d_6_*): δ = 0.88 (m, 3H, CH_3_), 1.85-1.88 (m, 2H, CH_2_), 4.16–4.18 (m, 2H, CH_2_), 7.66–7.73 (m, 2H, C_4_-H, C_5_–H), 9.09 (s, 1H, C_2_–H)

^19^F NMR (188 MHz, DMSO-*d_6_*): δ = −79.8.

1-butylimidazolium bis(trifluoromethylsulfonyl)imide (BIM-TFSI):

^1^H NMR (300 MHz, CDCl_3_): δ = 0.96 (m, 3H, CH_3_), 1.35 (m, 2H, CH_2_), 1.87 (m, 2H, CH_2_), 4.20 (m, 2H, CH_2_), 7.31–7.38 (m, 2H, C_4_-H, C_5_–H), 8.5 (m, 1H, C_2_–H), 11.18 (br s, 1H, NH)

^19^F NMR (188 MHz, DMSO-*d_6_*): δ = −79.97.

The structure of PILs used in this study is shown in Table 1.

### 2.3. Elaboration of the Porous Matrimid^®^ Membrane

The detailed procedure of the elaboration of the porous Matrimid^®^ membrane has already been described [10,26]. Briefly, the porous membrane was elaborated by the vapour induced phase separation method in the following way: Matrimid^®^ (14 wt.%) and PVP K90 (7 wt.%) were dissolved in NMP at 60 °C during 7 h under stirring to form a homogeneous solution. Then, the solution was left under stirring overnight to cool down to room temperature. The solution was poured on the flat glass plate by Doctor Blade (Elcometer, France). The glass plate with the casted solution was introduced into an oven with the controlled relative humidity (RH) and temperature for the certain time period. After that, the plate was immersed into a water bath to extract the porogen agent (PVP K90) and the solvent (NMP) and to peel off the membrane. Finally, the porous Matrimid^®^ membranes were washed in ethanol/water (50/50 *v/v*) mixture and dried at 90 °C overnight. The free-standing porous membranes with the thickness of about 180 µm were obtained by this method.

### 2.4. Preparation of the Composite Matrimid^®^/PIL Membrane

The porous Matrimid^®^ membranes (3.5 × 3.5 cm) were immersed into 10 g of PIL and left for 24 h at 60 °C for the correct incorporation of ionic liquid. Then, the solution was slowly cooled down to room temperature and the membranes were removed from ionic liquid. The PIL excess on the membrane surface was wiped out with a tissue. The PIL weight content in the composite membrane was in the range of 72 ± 1% for MIM-TFSI, 66 ± 1% for EIM-TFSI, 64 ± 1% for PIM-TFSI, and 63 ± 1% in the case of BIM-TFSI.

### 2.5. Characterization Methods

The morphology of the composite membranes was characterized by the scanning electron microscope (Carl Zeiss EVO^®^ 40 EP, Oberkochen, Germany) at the accelerating voltage of 15 kV. Before analysis, the membranes were cryofractured in liquid N_2_ to observe the cross-section. Both surfaces and the cross-section of the membranes were fixed on the carbon scotch and carbon coated.

The thermal stability of PILs and the composite Matrimid^®^/PIL membranes was analyzed by the thermogravimetrical analysis (TGA) using the Q500 analyzer from TA Instruments (New Castle, CA, USA) with a N_2_ flow rate of 90 mL/min. The experiments were realized on the samples of about 10 mg at the heating rate of 10 °C/min from 30 °C to 650 °C. The melting point, glass transition temperature (*T_g_*) and heat capacity of synthesized PILs were measured using the Netzsch DSC 204 differential scanning calorimeter (DSC) equipped with a liquid nitrogen cooling system. Each sample with the average weight of 15 to 20 mg was sealed in the aluminium pan, cooled down to −130 °C, and heated to 100 °C at the rate of 10 °C/min.

The mechanical characterization of the composite Matrimid^®^/PIL membranes was carried out by the Instron 5543 machine at a cross-head speed of 1 mm/min and 25 ± 1 °C. For reproducibility, five samples were analyzed for each membrane.

Water vapour sorption kinetic measurements were made at 25 ± 1 °C using the microbalance IGAsorp (Intelligent Gravimetric Analyzer Sorption moisture, Hiden Isochema Limited, UK). The sample time mass evolution is recorded when exposed to the atmosphere of a specific RH. The humidity is generated by mixing dry and saturated vapour gas flows in the correct proportions using gas flowmeters and a high precision hydrosensor. PILs were dried previously over P_2_O_5_ during a few days to remove the water, if any. Then, the PIL sample was loaded and equilibrated at 0% RH by the continuous flow of dry nitrogen, when equilibrium was reached, RH is changed from 0% to 95%. The water content was determined gravimetrically by the sample weighting until a constant weight was reached for each RH value, thus obtaining the maximum water content value at a given RH. Several mathematical models were used to fit the water vapour sorption data and provide additional thermodynamic information about the studied systems. The model’s parameters were calculated by fitting the water vapour sorption isotherm curves using Table Curve 2D software (version 5.01.01).

The Fourier-transform infrared (FTIR) spectra of PILs and the composite membranes were recorded in the range 650–4000 cm^−1^ at 228 scans per spectrum at 4 cm^−1^ resolution using the Nicolet Avatar 360 (Thermo Fisher, France) FTIR spectrometer in attenuated total reflectance (ATR) mode (equipped with a diamond crystal).

The proton conductivity measurements of the composite Matrimid^®^/PIL membranes and pure PILs were conducted by the two-probe method at 0.1, 1, and 10 kHz with the help of the immitance meter E7-14 in the temperature range 20–160 °C. Prior to the measurement, the sample was thermally equilibrated at every temperature in a temperature-controlled chamber. Then, the sample was kept for some period of time until the resistance became a constant value. The determination of the complex electrical conductivity σ*= *σ’* + *iσ”* was performed with the help of the impedance spectrometer Solartron SI 1260 in the frequency range from 10^−1^ to 10^6^ Hz. The proton conductivity σ was calculated as follows:

(1) $$\sigma =\frac{l}{RA}$$

where *l*, *A*, and *R* are the membrane thickness, membrane cross sectional area, and membrane resistance obtained from complex impedance plots, respectively.

## 3. Results and Discussion

### 3.1. Physico-Chemical Properties of PILs

Four new PILs based on the TFSI^-^ ion (Table 1) were easily prepared in high quality, and one of them (MIM-TFSI) was solid at 25 °C. The structures of all prepared PILs were confirmed by the ^1^H and ^19^F NMR analysis (see Section 2.2). The physico-chemical properties of these PILs were characterized by the DSC, TGA, infrared spectroscopy, and ionic conductivity. Some of the data are gathered in Table 1.

**Figure 1:**
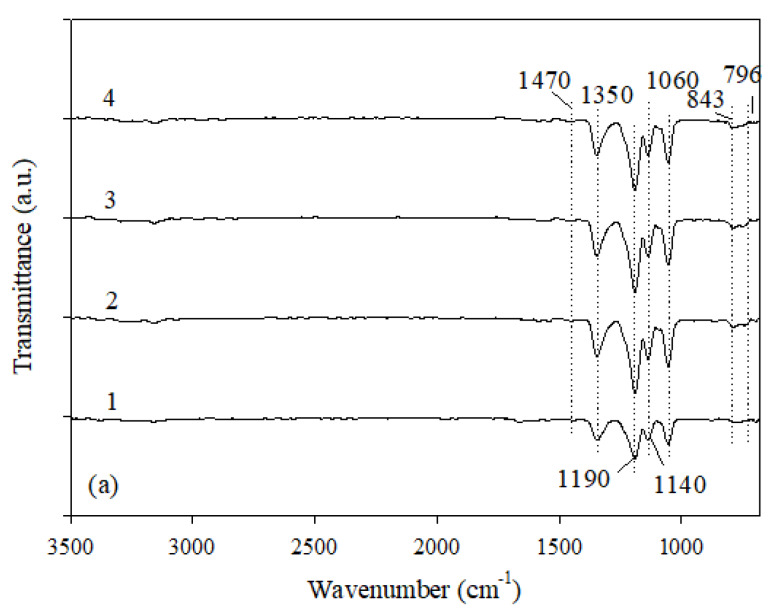

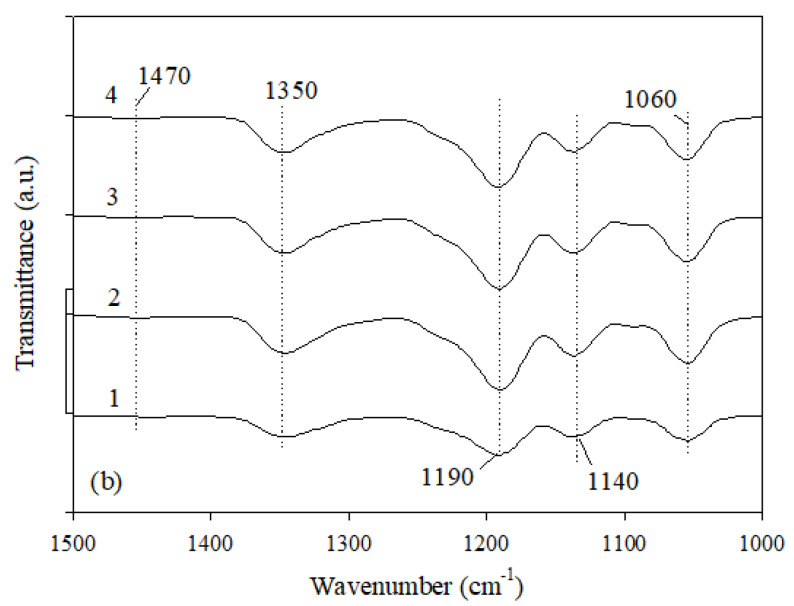
FTIR spectra of synthesized ionic liquids in the range of (**a**) 680–3500 cm^−1^ and (**b**) 1000–1500 cm^−1^: (1) MIM-TFSI, (2) EIM-TFSI, (3) PIM-TFSI and (4) BIM-TFSI.

The Fourier transform infrared spectroscopy gives very valuable information about the structure of a material. That is why the FTIR analysis was used to identify the PIL structure. The FTIR spectra for synthesized PILs are shown in Figure 1. The results of the FTIR analysis are very similar for all PILs. The bands at 1350 cm^−1^ and at 1190 cm^−1^ are assigned to the in-phase υ(SO_2_) vibrations and to the symmetric stretching vibration of SO_2_, respectively [29,30]. The band observed at 796 cm^−1^ and assigned to the antisymmetric vibrations υ(SNS) of the TFSI anion is, in fact, a vibration mode corresponding to the expansion and contraction of the whole anion [29,31]. The bands at about 1470 cm^−1^ and 843 cm^−1^ correspond to the asymmetric CH_2_ bending and rocking [31] and C–F vibration [32]. Besides, the stretching vibration of S=O is observed at 1060 cm^−1^ and the C–C stretching of the alkyl chain at 1140 cm^−1^ (Figure 1).

The solid-liquid phase transition behaviour of PILs was examined using the DSC analysis. At room temperature, MIM-TFSI is a crystalline solid, whereas the other three PILs (EIM-TFSI, PIM-TFSI, and BIM-TFSI) are liquids. The determined melting point for solid MIM-TFSI (52.8 °C, Table 1) agrees well with the literature data [33]. The variance of the melting point *T_m_* with the alkyl side chain for PILs follows a tendency similar to the one observed for other ionic liquids [34]. The presence of an asymmetrical structure generally provides less efficient ion-ion packing than in the case of a symmetric structure and, thus, a lower crystalline order and decreases *T_m_*. The DSC thermograms of liquid PILs (not shown) revealed that the glass transition temperature (*T_g_*) was equal to −82.3, −83.6, and −85.4 °C for PIM-TFSI, EIM-TFSI, and BIM-TFSI, respectively (Table 1). It is noted that *T_g_* of ionic liquids is manly governed by (1) the cation nature, including the ion size and polarity, and (2) the anion nature, including the ion size, flexibility and the extent of charge distribution [16,19,20,30,33,35]. One can note that BIM-TFSI has the lowest *T_g_* value (Table 1), which is the consequence of the PIL structure effect and can be attributed to lower Van der Waals interactions due to the longer alkyl chain length (−C_4_H_9_), compared to other PILs, which results in the easier segmental motion. Since *T_g_* is a qualitative measure of the ion mobility in ionic liquids [35,36], the obtained values testify to the high mobility of all these PILs and, thus, to their low viscosity. EIM-based ionic liquids usually show the lowest viscosity independently of the anion, which would be attributable to their nearly flat shape and the good charge distribution of the aromatic imidazolium ring [33,35]. The rotational freedom of molecules will be reduced with any longer and bulkier side chain on the imidazolium cation, that will lead to a higher viscosity. Indeed, the study performed with 1-*n*-3-methylimidazolium-TFSI ionic liquids has shown that the lowest viscosity is observed for 1-butyl-3-methylimidazolium-TFSI (49.9 mPa·s) compared to 1-hexyl-3-methylimidazolium-TFSI and 1-octyl-3-methylimidazolium-TFSI—69.7 and 90.0 mPa·s, respectively [37]. Therefore, one can suppose that the viscosity for synthesized PILs will increase in the following order: EIM < PIM < BIM.

During the heating from −100 to 100 °C, a sharp endothermic peak corresponding to the melting temperature (*T_m_*) was observed at 10.8 °C and 12.2 °C for PIM-TFSI and EIM-TFSI, respectively (Table 1). The coexistence of *T_g_* and *T_m_* indicates the relatively slow crystallization kinetics for these PILs, which has been widely observed in other ionic liquids [38].

The main advantages of ionic liquids are their non-flammability and high thermal stability, especially in comparison with the conventional electrolytes. Besides, the good thermal stability of a material is a crucial property when considering its application in the electrochemistry devices. The thermal stability of synthesized PILs was evaluated using the TGA measurements at the inert nitrogen atmosphere (Figure S1). As can be seen from Figure S1a, all synthesized PILs exhibit the high thermal stability (more than 280 °C) and similar TGA tendency. The decomposition temperature (*T_d_*) was defined as the temperature of the 1% weight loss. The maximum decomposition temperature (*T_max_*) is the point at which the maximum mass loss rate is observed, corresponding to the peak of derivative TGA (DTG curve, Figure S1b). The determined *T_d_* and *T_max_* values are gathered in Table 1. All studied PILs are thermally stable up to 300 °C followed by constant weight loss up to 430 and 450 °C, when they are decomposed completely (Figure S1).

The one-step degradation suggests the rapid backbone degradation that is followed by the onset of the weight loss. The observed peak on the DTG curves indicates the decomposition of the main chains of ionic liquid (Figure S1b). It can be seen that there is a general decrease in the thermal stability as the alkyl chain length increases (Table 1). It is found that MIM-TFSI is thermally the most stable, as it has the shortest alkyl chain length (CH_3_-). The decrease in the weight loss onset with the alkyl chain length suggests a degradation process similar to the Hofmann elimination for ammonium-containing species [39]. It can be stated that the thermal stability of PILs with the bis(trifluoromethylsulfonyl)imide anion is high and comparable with that of ionic liquids with other weakly coordinating anions, such as tris(pentafluoroethyl)trifluorophosphate [40] or bis(bis(pentafluoroehtyl)phosphinyl)imide anions [41]. The same behaviour was observed for other ionic liquids with the same (TFSI) anion [30,31]. The obtained results indicate that PILs have sufficient thermal stability to be used as the proton carriers in PEMFCs even at high temperatures (T > 130 °C).

**Figure 2:**
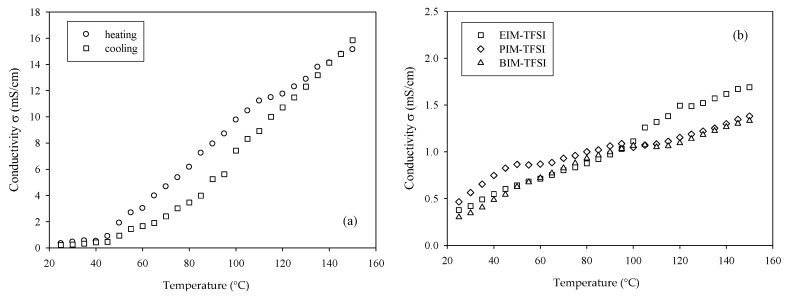
Ionic conductivity of synthesized PILs: (**a**) MIM-TFSI and (**b**) EIM-TFSI, PIM-TFSI, and BIM-TFSI during the heating cycle.

The temperature dependent ionic conductivity of synthesized PILs from 25 to 150 °C is shown in Figure 2. The conductivity was determined using the bulk resistance from complex impedance spectra according to Equation (1). Taking into account the influence of the PIL cationic structure on the conductivity, it is very important to carefully discuss the conductivity behaviour according to the experimental results. To begin, the conductivity increases practically linearly with increasing the testing temperature for all synthesized PILs due to their high ionic and thermal stability (Figure 2). However, a slightly different behaviour was observed for MIM-TFSI as it was the only PIL in the solid state (Figure 2a). As the melting of MIM-TFSI occurs in the measurement range (~53 °C, Table 1), the measured conductivity increases significantly due to the melting of this PIL, i.e., the ionic mobility increases significantly after ~53 °C.

It is known that the structural changes of a material may strongly modify its properties. It is found that in the solid phase the TFSI anion exists mainly in its trans conformation, while when melting both cis and trans conformers are present [32]. Furthermore, the ion pairs are most likely formed in the liquid phase of PIL [42]. In this case, the ions are linked by the hydrogen bonds through the N–H bond of the imidazolium ring and the oxygen atoms of the TFSI anion. The formation of such hydrogen bonding will significantly affect the proton transfer rate. That is why the conductivity increase by a factor of about 100 in the case of MIM-TFSI (0.34 and 15.1 mS/cm at 25 and 150 °C, respectively, Figure 2a) may be caused by the existence of the ion pairs which ensure more effective proton transfer pathways.

It is shown that the main proton transport mechanism that contributes to the proton conductivity in the case of ionic liquids is the Grotthuss mechanism, where the protons diffuse through the hydrogen bonds network [12,14,21]. It also appears that the conductivity of MIM-TFSI is more temperature sensitive than that of other PILs as a larger variation in conductivity is observed with the temperature increase (Figure 2a). The conductivity values obtained for other 1-alkylimidazolium PILs were found to be rather close to each other and almost one order of magnitude lower as compared with MIM-TFSI (Figure 2). Such behaviour may be explained by the lower mobility of the protonated imidazolium cations with the longer alkyl chain and, thus, by the ionic transport rate decrease. For example, at a given temperature, the conductivity of EIM-TFSI was always slightly higher than that of BIM-TFSI (Table 1 and Figure 2b). As the conductivity depends strongly on the mobility of the charge carriers, such behaviour is likely due to the higher viscosity caused by the stronger van der Waals interactions between the longer alkyl chains. Additionally, a longer alkyl chain takes a larger volume fraction of the neutral hydrocarbon part of the organic cation. The longer alkyl chain increases the proportion of hydrocarbons to fluorinated carbons in the composition of the PILs. Moreover, the increasing of temperature usually decreases the viscosity of PILs [30,37]. In our case, the close conductivity values for PILs in a liquid state mean that the length of the alkyl chain does not have a significant influence on the ionic conductivity.

The conductivity was found to increase with the temperature increasing. It means that the charge transfer between ions is prone to conduction once the attractive interaction in PILs becomes weaker with the temperature increase. Besides, practically negligible variation of conductivity was found between the heating and cooling temperature cycles (example of MIM-TFSI in Figure 2a) testifying to the high thermal stability of conductivity in the studied temperature range (from 25 to 150 °C) and, thus, to the potential application of these PILs in the middle and high temperature PEMFCs.

**Figure 3:**
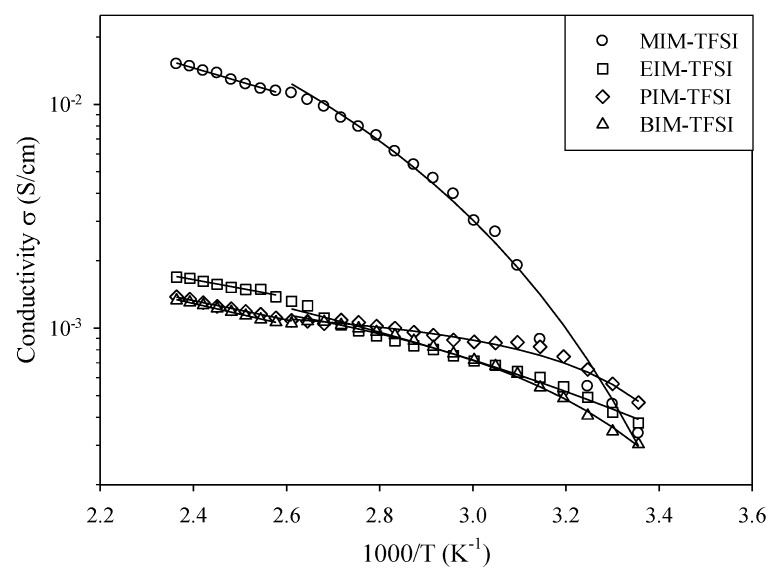
Temperature dependence of the conductivity for synthesized PILs. Solid lines are the best fit of the Arrhenius (Equation (2)) and VFT (Equation (4)) equations.

At high temperatures (starting from ~110 °C), the PIL conductivity shows a linear Arrhenius dependence as a function of the reciprocal temperature (Figure 3):

(2) $$\sigma =\sigma_{0}\;\exp(-\frac{E_{a}}{kT})$$

where *σ_0_* is the pre-exponential factor, *E_a_* is the activation energy, *k* is the Boltzmann’s constant, and *T* is the temperature in K. However, in the middle temperature range, the conductivity is non-Arrhenius as an upper concave curve is seen in Figure 3. Therefore, the conduction process can be well described by an empirical Vogel-Fulcher-Tamman (VFT) model as it is expected for the systems, in which the conductivity is essentially governed by viscosity. The VFT equation can be written as follows:

(3) $$\sigma =A\;\exp(-\frac{B}{T-T_{0}})$$

where *A* is the fitting parameter related to the concentration of the mobile charges, *B* is the empirical parameter accounting for the deviation of linearity (i.e., the more is curve dependence, the lower is *B*), *T_0_* is the Vogel temperature defining the low-temperature limit of the conductivity curves, and it is the temperature, at which the ion mobility approaches zero and the relaxation time extrapolates to infinity [43]. Besides, *B* is inversely proportional to the liquid fragility. This property is a measure of the sensitivity of the liquid structure to the temperature changes. Vila et al. have related the fitting parameters of the VFT equation with the Arrhenius equation assuming *A* = *σ_0_* and *B* = *E_a_/k* [44]. So, the final version of the VFT-type equation may be written as:

(4) $$\sigma =\sigma_{0}\;\exp(-\frac{E_{a}}{k(T-T_{0})})$$

where *σ_0_* is the maximum conductivity (if the temperature is infinity and assuming no degradation) and is proportional to the number of the charge carriers and *k* is the Boltzmann’s constant.

The best fit parameters estimated according to Equations (2) and (4) of ln *σ* versus 1/*T* data for synthesized PILs are gathered in Table 2. The number of the charge carriers (i.e., *σ_0_* value in the VFT equation (Equation (4)) decreases with increasing the alkyl chain length on the imidazolium ring. This may be the result of the ion pairs or ion clusters in pure PIL. Consequently, the alkyl chain length increase also reduces the glass transition temperature *T_g_* (Table 1) indicating higher ion mobility and decreases the activation energy *E_a_* (Table 2). The VFT activation energy values confirm well that the ion-dipole and hydrogen bond interactions are stronger for PILs with the shorter alkyl chain length. These stronger interactions explain the higher activation energy *E_a_* for the charge transport through PIL. At high temperatures, the slope of the conductivity temperature dependency appears to diminish for all studied PILs, as the conductivity value tends to a constant value (Figure 3). So, the temperature dependence of conductivity at higher temperatures (starting from ~110 °C) was fitted by the linear Arrhenius equation (Equation (2)). In this case, the activation energy *E_a_* increases with the alkyl chain length from 1-ethylimidazolium (0.076 keV) to 1-propylimidazolium (0.085 keV) and then to 1-butylimidazolium (0.095 keV). Such an increase is well agreed with the cation structure of PIL (Table 1) as better ion mobility and lower viscosity are observed for PILs with the short alkyl chain length.

The use of the VFT relation for the temperature dependence of the ionic conductivity is attributed to a strong coupling of ion transport and material segmental motions. The proton conduction occurs according to two mechanisms, namely, the proton hopping (Grotthuss mechanism) and the Einstein transport (vehicle mechanism). Generally, a Grotthuss mechanism is proposed to explain data for other PILs [11,12,21,45]. The decrease of the activation energy values with increasing the alkyl chain length for the Arrhenius and VFT parts is observed. This indicates the easier ionic transport in PIL. The thermal stability of conductivity as well as the high values of conductivity are of prime importance when considering these PILs as a proton carrier in the middle and high temperature PEMFCs.

It is very important that the amount of impurities in PILs is minimized because they can alter some chemical and physical properties. The most common impurity is water, which can be absorbed by ionic liquids when they are exposed to the moist air. Even if PILs with the alkyl groups containing more than three carbon atoms do not mix with water [12], they still may retain some water molecules. Moreover, it is shown that the alkyl chain on the cation reacts differently with the water molecules compared to the cation ring hydrogen [12,46,47]. Besides, the characterization of the liquid-liquid equilibrium (LLE) in the system containing PIL and water is important especially for evaluating ionic liquids as candidates for the fuel cell application, where the water molecules are the reaction product.

The water sorption isotherms of PILs containing the TFSI anion and different alkyl groups were studied by exposing them to the environment containing the different RH at 25 °C. For this purpose, the dynamic gravimetric technique was applied, which involves the use of the saturated water vapours to maintain a fixed RH when the equilibrium is reached. That was done in the controlled atmosphere chamber. The mass transfer between ionic liquid and the ambient atmosphere is assured by the natural diffusion of the water vapours. It should be noted that there was no mechanical stirring during the whole sorption process that prevented the water molecules from forming the percolation network inside PIL. The amount of sorbed water was measured at different time intervals and the equilibrium water content *C_W_* (in mmol of water/g of dry sample) was calculated at each RH according to the following equation:

(5) $$C_{w}=1000\frac{m_{eq}-m_{0}}{18m_{0}}$$

where *m_eq_* is the equilibrium mass at a given RH and *m_0_* is the dry mass.

**Figure 4:**
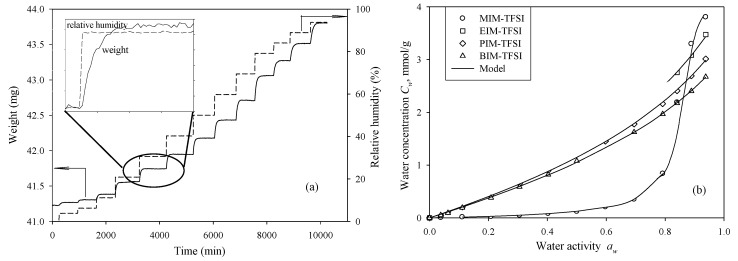
(**a**) Experimental sorption data and (**b**) water vapour sorption isotherms of studied PILs.

At the beginning of the exposure, ionic liquid absorbed water relatively fast and reached a saturation level after about 200 min (Figure 4a). Besides, the sorption equilibrium (saturation level) was achieved much faster for PILs containing shorter alkyl chains. For example, at 11% RH the equilibrium state was reached after 200, 250, and 513 min for EIM-TFSI, PIM-TFSI, and BIM-TFSI, respectively.

The water vapour sorption isotherm data (the equilibrium water content *C_w_*) were plotted against the RH reached (Figure 4b). As one can notice, the water molecules involved in the water sorption show a similar behaviour in all studied PILs, as all concentration isotherms are not-linear, i.e., the sorption isotherm demonstrates the concurrent increase in the *C_w_* value with the increase in the RH value (Figure 4b). A non-uniform distribution of sorption site energies across the material surface generally causes a non-linear isotherms shape. In our case the isotherm in the form of exponential shaped curve reflects Type III, according to the BET classification isotherms characteristics [48].

Figure 4b shows that only small amounts of water are sorbed by studied PILs, as the highest *C_w_* value reached at 95% RH is 3.8 mmol/g, which corresponds to the water mass fraction equal to ca. 6.8%. Such a result means that the hydrogen in 1-alkylimidazolium-TFSI enhances the hydrogen-bonding interaction of PIL-PIL and hence prevents the hydrogen-bonding interaction of PILs-water. It has been shown in the literature that the water sorption of ionic liquids is mostly governed by the anion nature [30,33,35,38,40,41,42,46,47,49,50,51,52]. For example, Tran et al. revealed that the higher quantity of water absorbed by ionic liquids indicated a stronger water-ionic liquid anion interaction [51]. The TFSI anion is known to be “hydrophobic” due to the greater opportunity for van der Waals interactions with water or alcohols compared to other anions, for example, PF_6_^-^ [49]. The PF_6_^-^ anion has a greater charge density than TFSI because it is smaller, so it can have stronger Coulombic or hydrogen-bonding interactions, which are very important in the case of water. Moreover, the increasing of the alkyl chain length of PILs results in the greater hydrophobicity, which may be translated to the stronger sorption via van der Waals force [46,53,54]. Therefore, the phase behaviour is the result of several competing interactions in the system, that take into account not only the composition of ionic liquid, but also the surrounding atmosphere.

A distinct sorption profile is observed for MIM-TFSI (Figure 4b) due to the fact that it is the only PIL in the solid state at room temperature. In this case the equilibrium water content *C_w_* is very low and increases only slightly with the increase of *a_w_* up to ~0.6. However, the significant increase of the *C_w_* value is observed at *a_w_* = 0.85 (Figure 4b). The plausible explanation is that at low RH the cation-anion interaction dominates the hygroscopicity. However, the higher RH (*a_w_* > 0.85) leads to higher quantity of the sorbed water molecules and, thus, to the greater interaction between the cation-water and anion-water, thus producing more hygroscopicity.

It is found that the maximum equilibrium water concentration *C_w_* decreases with increasing the cation alkyl chain length for the studied PIL-water systems (Figure 4b). Such tendency is expectable since the non-polar character of the alkyl groups increases with the number of carbons. Similar behaviour is also observed in other liquid systems [33,46,47,49,50,53,54].

Despite the large number of published articles, there is no appropriate model explicitly suitable for the phase equilibrium of ionic liquids. So, various models of LLE can be used. In general, LLE data are accompanied by correlations based on the excess Gibbs energy models, such as Wilson, Non-Random Two-Liquid (NRTL), UNIversal QUAsiChemical (UNIQUAC), electrolyte NRTL, modified NRTL, etc. [10,37,44,49,54,55,56,57]. The concept of the local composition is based on the fact that the system composition in the neighbourhood of a given molecule is not the same as the “bulk” composition because of the intermolecular forces. Data are commonly correlated by minimizing an objective function based on the squared differences between calculated and experimental compositions. Despite the fact that most of the Gibbs energy models available in the literature are used for non-electrolytes, they have been widely applied in modelling both the LLE and vapour-liquid equilibrium (VLE) in a variety of systems containing ionic liquids [44,54,55,56,57]. In the present study, the NRTL model is used as it is applicable to partially miscible systems and takes into account the effect of the temperature on LLE. It should be noted that the vapour pressure of ionic liquids is so low that the vapour phase is composed only of the other component (water in our case):

(6) $$P=P_{i}^{sat}\chi_{i}\gamma_{i}$$

where *P_i_^sat^* is the vapour pressure of the pure component (water) at the system temperature, *χ_i_* represents the mole fraction of component *i* in the liquid phase and *γ_i_* is the activity coefficient. In the NRTL thermodynamic model the activity coefficients are calculated as follows:

(7) $$\begin{array}{l} \ln\gamma_{i}=X_{j}^{2}\left({\left(\frac{G_{ji}}{X_{i}+X_{j}G_{ji}}\right)}^{2}\tau_{ji}+\frac{G_{ij}\tau_{ij}}{{\left(X_{j}+X_{i}G_{ij}\right)}^{2}}\right)\\ \ln\gamma_{j}=X_{i}^{2}\left({\left(\frac{G_{ij}}{X_{j}+X_{i}G_{ij}}\right)}^{2}\tau_{ij}+\frac{G_{ji}\tau_{ji}}{{\left(X_{i}+X_{j}G_{ji}\right)}^{2}}\right) \end{array}$$

with

$$G_{ij}=\exp(-\alpha_{ij}\tau_{ij})$$

$$\tau_{ij}=\frac{g_{ij}-g_{jj}}{RT}=\frac{\Delta g_{ij}}{RT}$$

$$\tau_{ji}=\frac{g_{ji}-g_{ii}}{RT}=\frac{\Delta g_{ji}}{RT}$$

where *χ* represents the mole fraction, *g_ij_* is an energy parameter characterizing the interaction of species *i* and *j*, *R* is the gas constant, *T* is the absolute temperature, and the parameter α*_ij_* = α*_ji_* is related to the non-randomness in the mixture; when *α_ij_* is zero, the mixture is completely random, or ideal solution. Despite the fact that *α_ij_* is an adjustable parameter, it can also be considered as fixed, usually between 0.20 and 0.47 [54,55,56,57], so the model will have only two adjustable parameters per binary system. The binary interaction parameters Δ*g_ij_* (or the equivalent *τ_ij_*) are calculated from the experimental data. The criterion for the fit quality is the mean relative deviation modulus (*E*):

(8) $$E=\frac{100}{N}\sum_{i=1}^{N}\left|\frac{P_{i,\exp}-P_{i}^{model}}{P_{i,\exp}}\right|$$

where *P_i,exp_* is the *i*^th^ experimental point value, *P_i_^model^* is the *i*^th^ model predicted value, and *N* is the number of data points. It is generally considered that *E* values below 10% indicate an adequate fit for the practical use [58].

The values of the NRTL binary interaction parameters obtained for each studied PIL-water system are shown in Table 3. The goodness of fit between the experimental and NRTL calculated (Equation (7)) parameters was evaluated through the mean relative deviation modulus *E* (Equation (8)) and the corresponding values of *E* for each system are also added in Table 3. As one can see, the *E* values less than 10% indicate that the NRTL correlation is able to represent the sorption experimental data very well. A little higher *α* value for MIM-TFSI (*α* = 0.45, Table 3) may be attributed to the solid state of this PIL compared to the liquid state of the other PILS (*α* = 0.4). It is known that in the ideal case of the random system *α* is equal to 0 [49,56]. The highest value of *α* (0.45) means that the water molecules are less randomly dispersed in MIM-TFSI. The *τ* values may be interpreted in terms of the differences in the interaction energies between the water molecules and PIL. The cation/anion interaction should be the strongest one, followed by the anion/solvent and cation/solvent interactions, and the solvent/solvent interaction should be the weakest [51,52]. This means that one would expect *τ_21_* ≤ 0; *τ_12_* ≥ 0; |*τ_21_*| << |*τ_12_*| (index 1 corresponds to the water molecules and index 2—to PIL). As seen from Table 3, this supposition is well confirmed for all PILs in the liquid state at 25 °C (i.e., except MIM-TFSI). Besides, one can see that the obtained *τ* values are rather similar for these PILs.

In order to better understand the water behaviour in PILs, the obtained water sorption isotherms were also analysed by the modified D’Arcy and Watt model, which not only gives information about the ion-water interactions in the material containing the ionic groups, but also permits to distinguish different types of the sorption sites [59]:

(9) $$C_{w}=\frac{K_{1}K_{2}a}{1+K_{1}a}+K_{3}a+\frac{K_{4}K_{5}a}{1-K_{4}a}$$

where *a* is the water activity, and *K_i_* are different parameters related to the bond energy (*K_1_* and *K_4_*), to the number of the active sites involved in the Langmuir sorption (*K_2_*), to the water solubility in the material (Henry’s law) (*K_3_*), and to the capacity of the water molecules to form multilayer (*K_5_*). In this model, the first term refers to the Langmuir-type sorption sites (primary sorption sites), the second term relates to the weak sorption sites, and the third term to the multilayer formation. The experimental sorption isotherms of PILs were fitted according to this model by minimizing the average relative deviation modulus *E* (Equation (8)) (Figure 4b and Table 4).

The predicted results of the D’Arcy and Watt model are in good agreement with the experimental data; the *E* value is less than 7% in all cases (Table 4). As one can see from Figure 4b, there is no flattish portion in all isotherms, indicating that the monolayer formation (Langmuir’s sorption) is missing. The very small (practically negligible) *K_1_* and *K_2_* values also indicate that there is a limited number of the sites for the specific bulk water sorption (Table 4). Chen et al. studied the atmospheric water sorption in 1-butyl-3-methyl-imidazolium acetate (BMIM-Ac) by means of the two-dimensional correlation infrared spectroscopy [46]. They revealed that the dynamic process of the water sorption could be divided into three parts: during the first 120 min only the bulk water sorption takes place due to the presence of the highly hydrophilic acetate anion; from 120 to 320 min, the bulk and surface sorption happens simultaneously, and from 320 min up to the steady state only the surface sorption occurs. As in our case, a rather hydrophobic TFSI anion is used [49,60] and the low concentration of the sorbed water at *a_w_* < 0.2 is not surprising. Indeed, the only place where the bulk water sorption could take place is the number 2 hydrogen on the imidazolium ring (Table 1) as it is highly acidic and able to form hydrogen-bonding with water [49]. In addition, the absorbed bulk water (if any) prohibits ionic liquid to sorb more water molecules into the bulk part. It may be caused by the film formation of the water molecules on the PIL surface [46,54] and the formed film would prevent the water from entering further into the PIL bulk. This again reflects the high hydrophobicity of PILs, which means that the water molecules tend to cluster together rather than interact with the ionic liquid molecules.

The *K_3_* value (Henry’s sorption) increases with the alkyl chain length increase (Table 4). This fact indicates that water starts to interact with the already absorbed water molecules by the hydrogen bonding. Additionally, the steric effect induced by the increase of the chain length would create more free space allowing the water molecules to be more dispersed. As the groups that are able to interact with the water molecules (i.e., anion and hydrogen on the cation ring) have already reacted, the aggregation phenomenon observed at the higher RH values, i.e., at *a_w_* > 0.7 (Figure 4b), would preferably occur at the PIL surface, assuming that the addition of the water molecules is more difficult inside PIL because of the strong interaction between the cation and anion. However, except the particular case of MIM-TFSI, the difference observed in the *K_5_* value (with a *K_5_* value significantly lower for BIM-TFSI in agreement with the PIL chemical structure (Table 1)) reveals that the water clustering is favoured in the case of ionic liquids with the shorter alkyl chain length. The increase of the *K_5_* values can be explained by a reduction in the number of the water clusters due to the changes of the alkyl chain length, i.e., its shortening.

In order to see the influence of the PIL state (either liquid or solid) on the water sorption process, the measurements were performed for MIM-TFSI at 60 °C, i.e., above its melting point (Table 1). As one can see from the obtained D’Arcy and Watt model parameters (Table 4), the significant increase of the *K_2_*, *K_3_*, and *K_5_* values is observed. This fact testifies to the greater number of the vacant sites for the specific bulk water sorption, to the easier interaction with the already sorbed water molecules by the hydrogen bonding and to the favoured water clustering observed for PIL in the liquid state.

The performed desorption measurements (not shown) have revealed that no hysteresis exists between the sorption and desorption cycles as the deviation of about 0.5% was observed between the values of *C_w_* measured during the water vapour sorption and desorption. The similar results were also found in the case of other ionic liquids [46,50,53]. Despite the fact that the water molecules are absorbed both in the bulk and at the free surface of PILs, the desorption results indicate that the water incorporation is essentially a reversible process, and the latter mechanism (absorption at the PIL surface) seems to be dominant.

**Figure 5:**
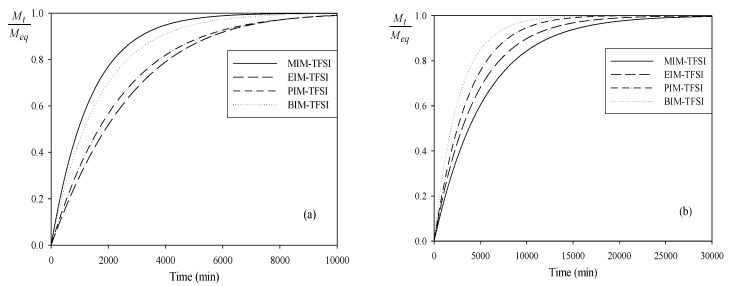
Influence of the RH value on the water uptake for studied PILs at 25 ± 1 °C: (**a**) 70% RH, (**b**) 94% RH.

In order to measure the capacity of the water molecules to move into PIL, the total mass sorption can be described by the mass uptake $\frac{M_{t}}{M_{eq}}=f(t),$ where *M_t_* and *M_eq_* are the quantity of the sorbed water at time *t* and at the equilibrium, respectively. For all studied PILs, the water sorption kinetic curves determined at two different RH levels (i.e., 70% and 94%) are presented in Figure 5. The choice of the RH value was based on the isotherms’ results (Figure 4b) in order to study the diffusion during the Henry’s sorption and the cluster formation. One can see that EIM-TFSI exhibited substantially slower kinetics than PIM-TFSI and BIM-TFSI whatever the RH value (comparing Figure 5a,b). This result can be explained by the water lower mobility. Thus, the cluster organisation takes more time, and the diffusion is slower. The highest *K_5_* value obtained for EIM-TFSI (1.585, Table 4) confirms this explanation well. The totally different diffusion behaviour for MIM-TFSI (i.e., the fastest at 70% RH and the slowest at 94% RH) may be explained by the solid state of this ionic liquid at 25 °C. In this case, it takes less time to attain the water sorption equilibrium at 70% RH than at 94% RH. As it can be seen from Figure 4b, the concentration of the sorbed water in this case is less than 1 mmol/g up to 80% RH. Thereafter, a sharp increase of the sorbed water concentration *C_w_* is revealed. This fact is attributed to the water clustering formation, which occurs at higher activity *a_w_*, when the water molecules exist in multi-molecular clusters rather than as single molecules. So, the highest water concentration *C_w_* = 3.81 mmol/g at 94% RH is observed for this PIL (Figure 4b).

### 3.2. Composite Matrimid^®^/PIL Membranes

The conductivity of the pure Matrimid^®^ film was about 10^−13^ S/cm. Therefore, in order to use this mechanically and thermally stable polymer in the field of fuel cells, its proton conductivity should be improved by increasing the amount of charged groups. That is why the proton conductive phase (i.e., PIL) was added into the polyimide film by swelling.

The composite Matrimid^®^/PIL membranes were prepared by the swelling of a porous polyimide film in the solution of a given PIL at 60 °C during 24 h. It was shown that this time period was sufficient for the complete impregnation of the polymer support [10]. The choice of 60 °C was based on the melting point of MIM-TFSI (Table 1). As the polyimide is an insulating polymer, the ionic conductivity is ensured only by the PIL presence. It is well known that the level of dispersion of the conducting phase in a polymer matrix, the so-called percolation threshold, has a crucial influence on the properties of the whole material. So, at first the presence of PIL in the porous polyimide film is confirmed by means of FTIR, TGA, mechanical, and SEM measurements. In each case the pure Matrimid^®^ film is used for comparison.

**Figure 6:**
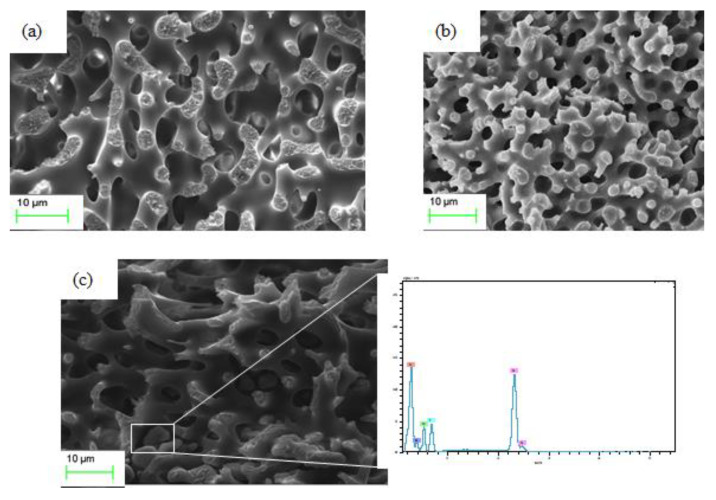
Cross-section SEM images of cryo-fractured membranes: (**a**) Matrimid^®^ film, (**b**) Matrimid^®^/BIM-TFSI, (**c**) Matrimid^®^/MIM-TFSI. Inset: EDX pattern.

Previously, it has been shown that the structure of the Matrimid^®^ film elaborated by the water vapour induced phase inversion method demonstrates a porosity at two levels [10,26]: the main (primary) symmetrical spongy and interconnected macroporous (from 10 up to 20 µm) structure on the surface and the secondary macroporous (less than 1 µm) structure within the dense part of the film (Figure 6a). The porosity ratio (the pore volume/the total film volume) of the film elaborated by this method is estimated to be 66% [10]. The high porosity and the interconnectivity of the pores allow the efficient introduction of PIL inside the polyimide films. The penetration of the ionic liquid inside the secondary macroporous structure is facilitated by the temperature increase up to 60 °C during the swelling procedure. In the case of all studied PIL the swelling degree is close to 63% to 72%.

High-resolution SEM images of the cross-section of the cryo-fractured composite Matrimid^®^/PIL membranes are found to be different from the neat Matrimid^®^ film when comparing Figure 6a with Figure 6b,c. The pure Matrimid^®^ film revealed the presence of the secondary open macroporous structure within the polymer phase, whereas after the incorporation of ionic liquid these pores were completely filled with PIL (Figure 6b,c). This result implies that the PIL swelling is complete. Energy dispersive X-ray (EDX) patterns of the membranes (surface and cross-section) were performed to detect the presence of the characteristic elements of PIL in the polyimide pores (Inset to Figure 6c). The presence of S and F peaks in the case of all studied composite membranes confirms the successful incorporation of PIL (Table 1) in the polyimide porous film. In addition, one can also distinguish the presence of MIM-TFSI in the membrane macropores (Figure 6c) as this ionic liquid is solid at room temperature. Due to the vacuum step during the SEM analysis, it is obvious that the primary macroporous structure (of higher pore size) is free from PIL.

**Figure 7:**
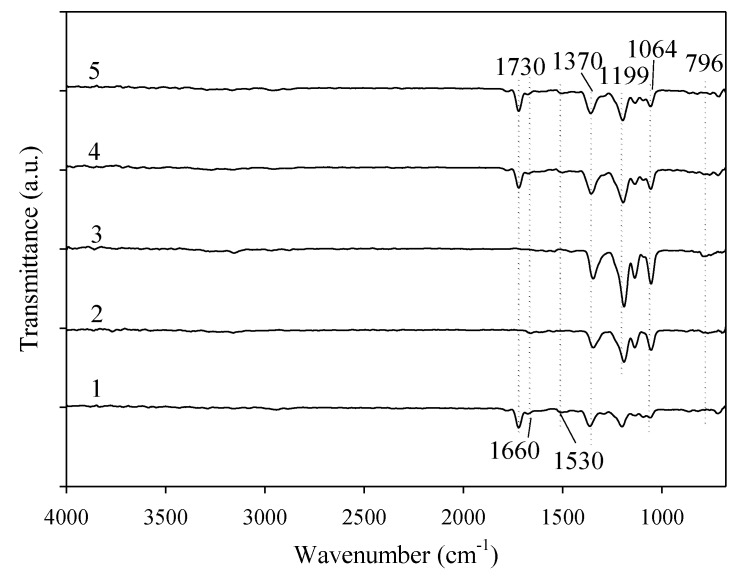
FTIR spectra of (1) Matrimid^®^ film, (2) MIM-TFSI, (3) BIM-TFSI, (4) Matrimid^®^/MIM-TFSI membrane, (5) Matrimid^®^/BIM-TFSI membrane.

In order to study the possible interactions between PIL and polyimide membrane, FTIR spectroscopic analysis was carried out. Figure 7 shows the comparative FTIR spectra of pure ionic liquid (BIM-TFSI and MIM-TFSI), the Matrimid^®^ film and the composite Matrimid^®^/PIL membranes. The spectra of pure Matrimid^®^ and the composite membranes show the typical peaks of the imide groups at 1730 cm^−1^ and 1370 cm^−1^ as well as the peaks corresponding to the amide groups at 1660 cm^−1^ and 1530 cm^−1^ [61]. In addition, the spectra of the Matrimid^®^/PIL membranes reveal a characteristic peak of the TFSI anion (SNS bond) at 796 cm^−1^ [29,31] testifying to the PIL presence inside the membrane. The slight shift of the bands corresponding to the stretching vibration of S=O (from 1060 cm^−1^ to 1065 cm^−1^), the symmetrical stretching (from 1190 cm^−1^ to 1203 cm^−1^) and the in-phase vibrations of SO_2_ (from 1350 cm^−1^ to 1365 cm^−1^) to a higher wavenumber region noticed in the case of the composite membranes (Figure 7) testifies to the polyimide interaction with PIL. Besides, the vibration of C=O group was noted to shift from 1724 cm^−1^ (in the case of the pure polyimide film) to 1726 cm^−1^ (for the composite membranes) (Figure 7). The positively charged cation of PIL can be electrostatically attracted to the negatively charged ion pair of electrons on the oxygen atoms of the carbonyl (C=O) groups in Matrimid^®^. The similar behaviour was observed in the case of 1-butyl-3-methylimidazolium trifluoromethanesulfonate with poly(ethyl methacrylate)/poly(vinylidene fluoride-*co*- hexafluoropropylene) films [62]. Besides, the shorter cation alkyl chain length is, the higher the FTIR position shift is observed testifying to the stronger coordination of ionic liquid with the polymer structure.

**Figure 8:**
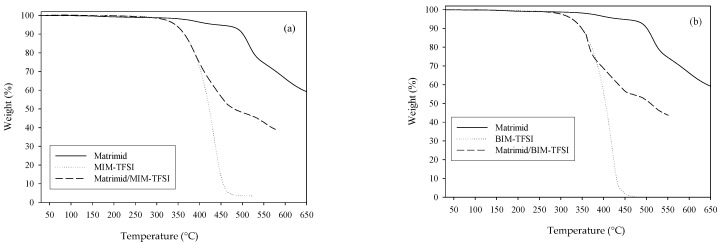
TGA curves of the composite Matrimid^®^/PIL membranes based on: (**a**) MIM-TFSI and (**b**) BIM-TFSI.

The TGA analysis was also performed to study the effect of the PIL loading on the thermal stability of the composite membranes. As it can be seen from Figure 8, there are two main weight losses in the case of the pure polymer film. The first (less than 5%) starts at about 360 °C and ends at about 520 °C and corresponds to the removal of the traces of a pore forming agent (polyvinylpyrrolidone) from the membrane. The second weight reduction in pure Matrimid^®^ is at 560 °C, that is the temperature at which the polymer chain degradation begins. Due to the high amount of added PIL (64% to 70%), the degradation of the composite membranes starts at the temperatures close to the degradation of pure PIL (Table 1 and Figure S1). The second weight loss in the case of the composite membranes is related to the decomposition of polyimide. Besides, the thermal degradation of the composite membranes based on MIM-TFSI starts at a higher temperature than the degradation of the BIM-TFSI based membrane (compare Figure 8a,b). The found *T_d_* is 290 °C for the Matrimid^®^/MIM-TFSI membrane and 261 °C for the Matrimid^®^/BIM-TFSI membrane. This result may be explained by higher electrostatic interactions between polyimide and ionic liquid based on the imidazolium cation with a shorter alkyl chain length, which delays the composite decomposition.

Mechanical stability of the composite membranes was measured, and the Young’s modulus values are gathered in Table 5. Mechanical robustness of the polymer membrane can play a critical role and prevents the short circuit between anode and cathode during the fuel cell operation. As one can see from the obtained results, incorporation of ionic liquid causes the increase of the Young’s modulus value—whatever the ionic liquid used is. This result is explained by possible physical interactions between the polymer matrix and ionic liquid (in our case, the imidazolium rings of ionic liquid).

**Figure 9:**
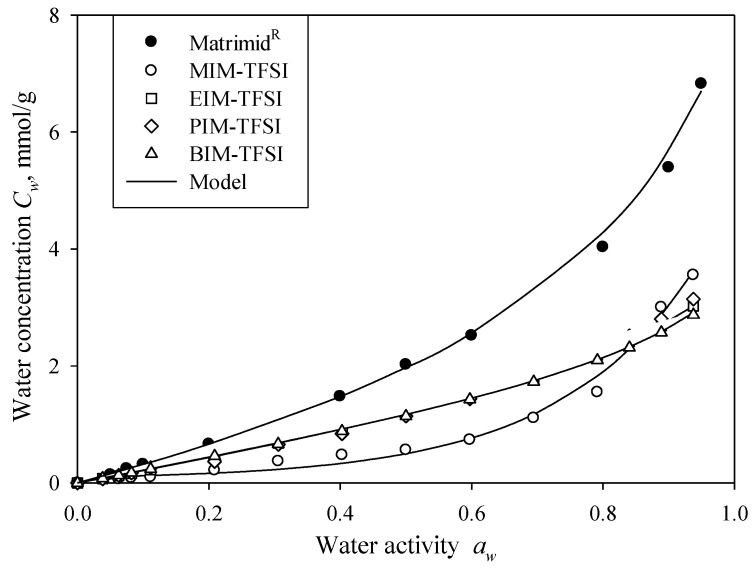
Water vapour sorption isotherms of the elaborated Matrimid^®^/PIL composite membranes at 25 ± 1 °C. Solid lines are the best fit of the D’Arcy and Watt model (Equation (9)).

The effect of the PIL presence on the water vapour sorption properties of the composite membrane based on Matrimid^®^ at 25 °C is shown in Figure 9. The sorption behaviour of the pure Matrimid^®^ membrane is given for comparison. The water vapour sorption isotherms are described by the D’Arcy and Watt model (Equation (9)) due to the physical meaning of the model parameters. The model parameters were obtained by fitting data points and gathered in Table 5. As in the case of pure PILs (Table 4), the predicted results of the D’Arcy and Watt model are in good agreement with the experimental data.

As one can see, a pure Matrimid^®^ membrane absorbs more water than composite membranes (Figure 9). At low activity, sorption is accompanied by a rather low adsorption process, followed by the random water dissolution in the membrane. That is why the Henry’s water concentration is much higher than the water adsorption on the Langmuir sites. This can be attributed to the large expansion of the effective sorption surface in the porous membrane, especially taking into account that the Matrimid^®^ porosity is equal to 66% [10]. Additionally, it is possible that the microcapillary condensation of the water molecules in the membrane pores is added to the Henry’s dissolution. Generally, at high water activity, the water molecules interact with the polymer matrix or/and with the other water molecules via the hydrogen bonds to form the clusters or agglomerates. In the case of Matrimid^®^, which possesses low hydrophilicity and some stiffness of the polymer backbone, the clustering of the water molecules is observed with a rather high capacity of the water molecules to form multilayer that is testified by the high *K_5_* value (Table 5).

In the case of the Matrimid^®^/PIL composite membranes, one can note that the equilibrium water concentration *C_w_* is very low compared to the pure Matrimid^®^ membrane whatever PIL is used (Figure 9). Moreover, the sorption capacity of the Matrimid^®^/PIL composite membranes is comparable with that for pure PILs (Figure 4b). As in the case of pure PILs, the membrane capacity to form the water clusters is decreasing with the PIL alkyl chain increasing (compare *K_5_* values, Table 5). This result can be explained by the fact that the composite membrane consists mainly of the PIL phase (~64% to 70%). Thus, taking into account all foregoing, one can say that the conductivity value of the composite membrane is dependent mostly on the PIL nature (i.e., its capacity to transfer the protons) than on its water sorption behaviour.

**Figure 10:**
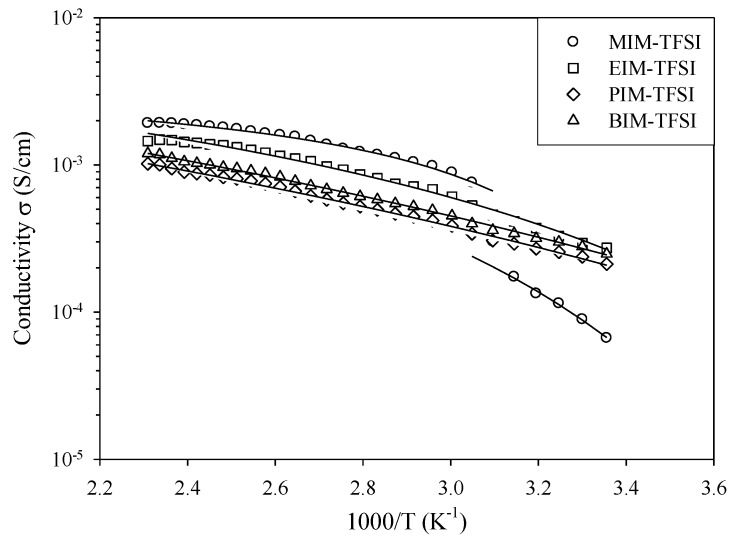
Temperature dependence of the conductivity of the composite Matrimid^®^/PIL membranes. Solid lines are the best fit of the VFT (Equation (4)) equation.

The temperature evolution of the ionic conductivity of the Matrimid^®^/PIL composite membranes measured using a two-probe method in the thickness direction is shown in Figure 10. The ionic conductivity of all composite membranes increases with the temperature increase. In any electrolyte, the conductivity value depends on the number density of the mobile ions and on the ionic mobility. Therefore, the observed conductivity increase with the temperature could be caused by the fact that PIL acts as both a strong ion carrier and as a plasticizer to enhance the segmental mobility of the polymer chains that makes the polyimide backbone more flexible to help the ion transfer.

Therefore, the conductivity of such composite membranes is significantly promoted by the temperature increasing. The proton conductivity of the composite membranes at 25 °C is in the range of 10^−5^ to 10^−4^ S/cm and, therefore, is much lower than the conductivity of fully hydrated Nafion^®^, which has a proton conductivity of 0.1 S/cm at 25 °C [63], but is comparable with the conductivity of polybenzimidazole (PBI) membrane, which is about 10^−5^ S/cm at 25 °C [64,65,66,67]. So, at 25 °C Nafion^®^ membrane has the highest performance. However, at higher temperatures (>80 °C), the Nafion^®^ performance degrades rapidly because of the dehydration phenomenon and the conductivity of ~10^−8^ S/cm or even less is obtained [1,2,63]. On the contrary, the conductivity of the PBI/PIL membranes increases with the temperature increase [65,66,67]. For example, the proton conductivity of 1.5 × 10^−3^ S/cm was obtained at 160 °C for the porous PBI membrane impregnated with 1-H-3-methylimidazolium bis(tri-fluoromethanesulfonyl)imide [64]. In our case, the conductivity of the composite Matrimid^®^/PIL membranes also continues to increase with the temperature rise up to 160 °C, reaching 10^−2^ to 10^−3^ S/cm (Figure 10). Besides, the low hysteresis of the heating and cooling curves of the temperature dependence of the proton conductivity (not shown here) indicates good thermal conductivity stability of the composite membranes, as well as a high retention of the PIL electrolyte inside the porous polymer matrix. These results confirm that the proton conduction in the composite membrane is due to the proton ionic carriers. Ionic liquid can provide continuous conducting channels by replacing water under the anhydrous conditions (at 100 to 150 °C). This result is of great interest, because an operating temperature of 120 to 150 °C is considered high enough to confer the benefits of a middle- and high-temperature operation. So, the elaborated Matrimid^®^/PIL membranes are promising candidates for the middle- and high-temperature fuel cell application.

Taking into account that PIL is a proton conducting medium, the proton conduction in the Matrimid^®^/PIL membranes occurs predominantly according to the Grotthuss mechanism. One can see that in the range from 25 to 150 °C, the conductivity evolution versus the reciprocal temperature is not linear (Figure 10), thus indicating that the proton conduction does not just follow Arrhenius-type shape, but also shows the VTF behaviour (Equation (4)). The conductivity parameters and activation energy values have been calculated from these dependences (Table 6). The activation energy values for all composite membranes range from ~30 to ~57 keV. These values may be considered as indicative of a structural diffusion mechanism and may confirm that the conduction in all studied composite membranes conforms to the Grotthuss mechanism [63]. On the basis of these results one can conclude that the interconnected porous structure of the porous polyimide matrix ensures a continuous proton conducting phase.

The TFSI anion from ionic liquid can interact with polyimide and displace the proton away from exchange sites (N-H) of Matrimid^®^. Thus, the ionic channels within the polymer host matrix will be formed enhancing the conductivity. Besides, as in the case of pure MIM-TFSI, the conductivity dependence of the composite membranes based on this PIL presents two regions (low-temperature and high-temperature) with the inflexion point at ~60 °C (i.e., the PIL melting point). This result confirms the presence of the charged species with a higher mobility at raised temperatures able to improve the proton conductivity. The calculated values of the activation energy for these two parts (Table 6) clearly indicate facilitated proton conduction in the high-temperature region (*E_a_* = 7.56 keV). The conductivity values of the composite membranes are slightly lower than the values for pristine PILs (comparing Figure 3 and Figure 10). The reason is that the presence of the proton insulating polymer matrix (even porous one) causes a strenuous ionic migration in the composite membrane as the conduction channels are formed only through the PIL cations.

The eventual loss of PIL from the membrane by blowing was determined by weighing the composite membranes before and after the conductivity measurements. The amount of PIL loss was estimated at ~5 wt.% whatever the ionic liquid used. Such low value indicates that PILs are well trapped in the porous structure of the membrane and that the composite membranes would retain all PIL during the fuel cell operation.

The best conductivity performance was measured for Matrimid^®^/MIM-TFSI membrane. In that case, the conductivity was the highest, which is attributed to the facilitated proton long-range transport. In order to have a deeper insight into the conduction mechanism, more experiments should be performed. However, at least, this study has shown that such composite membranes may be used in PEMFC operated at much higher temperatures compared to the Nafion^®^ membrane. This fact will lead to using PEMFCs with a higher CO tolerance, better reaction kinetics, and a simpler water management.

## 4. Conclusions

New PILs based on alkylimidazolium-TFSI were successfully synthesized and they have emerged as a promising protic solvent to replace the water molecules in fuel cells and to enable the operation under middle- and high- temperature conditions. The PILs water sorption isotherms were determined from the experimental sorption kinetic data and fitted using the D’Arcy and Watt and non-random two-liquid models. It was shown that the PIL conductivity was not affected significantly by the alkyl chain length. The water vapour sorption results testified to the low water sorption of pure PILs (less than 4 mmol/g) ensuring their high hydrophobic character. At the same time, the observed conductivity increasing with the temperature rising up to 160 °C revealed the possibility of preparing low cost composite membranes with high proton conductivity values.

The composite membranes based on the porous polyimide Matrimid^®^ membrane and PIL were elaborated by an impregnation process. The interaction and compatibility of two components (i.e., polymer and PIL) were investigated by the vibration spectroscopy (FTIR-ATR) and the scanning electron microscopy, respectively. The thermal analysis results showed an adequate thermal stability of the Matrimid^®^/PIL composite membranes, which is essential for the fuel cell application. FTIR characterization confirmed the presence of PIL inside the polyimide membrane. The measured activation energy values ranged from ~30 to ~57 keV confirming that the ionic conduction followed the Grotthuss mechanism. Although the elaborated composite membranes demonstrated good thermal properties and an improved proton conductivity stability as a function of temperature, their proton conductivity values are still not sufficient for using them at high temperature. Therefore, an optimization of the composite membrane (for example, the PIL content) as well as the measurements of the polarization curves under different conditions (i.e., temperature and gas composition) will be the subject of further research.

## Figures and Tables

**Table 1:** Physico-chemical properties of synthesized protic ionic liquids (PILs).

PIL	Cation	Anion	*T_g_*^a^ (°C)	*T_m_*^b^ (°C)	*T_d_*^c^ (°C)	*T_max_*^d^ (°C)	*σ*^e^ (mS/cm)
MIM-TFSI	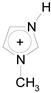	(CF_3_SO_2_)_2_N^-^	−	52.8	291	437	0.339
EIM-TFSI	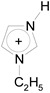	(CF_3_SO_2_)_2_N^-^	−83.6	12.2	285	421	0.377
PIM-TFSI	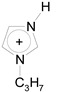	(CF_3_SO_2_)_2_N^-^	−82.3	10.8	285	429	0.466
BIM-TFSI	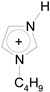	(CF_3_SO_2_)_2_N^-^	−85.4	−	268	422	0.303

^a^ Glass transition temperature. ^b^ Melting point. ^c^ Decomposition temperature. ^d^ Maximum decomposition temperature. ^e^ Ion conductivity at 25 °C.

**Table 2:** Fitted values of the ionic conductivity according to the Arrhenius (Equation (2)) and VFT (Equation (4)) equations.

PIL	Arrhenius Part		VFT Part		
	*σ_0_*, S/cm	*E_a_*, keV	*σ_0_*, S/cm	*T_0_*, K	*E_a_*, keV
MIM-TFSI	0.4154	0.120	0.2003	234.4	35.69
EIM-TFSI	0.0138	0.076	0.0073	164.2	33.71
PIM-TFSI	0.0144	0.085	0.0014	270.9	2.60
BIM-TFSI	0.0179	0.095	0.0025	248.2	9.14

**Table 3:** Binary interaction parameters and mean relative deviation modulus *E* of experimental phase equilibrium with the NRTL equation for the binary system (water (1)-PIL (2)).

PIL	*τ_12_*	*τ_21_*	*α*	*E,* %
MIM-TFSI	1.18	2.41	0.45	9.96
EIM-TFSI	4.59	−0.36	0.4	9.06
PIM-TFSI	4.99	−0.32	0.4	6.74
BIM-TFSI	4.30	−0.32	0.4	6.99

**Table 4:** The D’Arcy and Watt model parameters (Equation (9)) and the mean relative deviation modulus *E* (Equation (8)) for synthesized PILs.

PIL	Temperature, °C	Model’s Parameters					
		*K_1_*	*K_2_*	*K_3_*	*K_4_*	*K_5_*	*E*, %
MIM-TFSI	25	1.547 × 10^−6^	0.0050	0.002	1.134	0.095	6.3
	60	2.257 × 10^−8^	33.17	3.817	0.8548	1.346	3.9
EIM-TFSI	25	1.000 × 10^−6^	0.0901	1.007	0.6517	1.585	6.1
PIM-TFSI	25	4.135 × 10^−8^	0.0050	1.080	0.6520	1.255	5.0
BIM-TFSI	25	4.982 × 10^−6^	0.0089	1.145	0.6624	0.972	5.6

**Table 5:** The D’Arcy and Watt model parameters (Equation (9)), the mean relative deviation modulus *E* (Equation (8)) and the Young’s modulus values for the Matrimid^®^/PIL composite membranes.

Membrane	Model’s Parameters					*E*, %	Young’s Modulus, MPa
	K_1_	K_2_	K_3_	K_4_	K_5_		
Matrimid^®^	2.45 × 10^−8^	0.0195	1.734	0.788	1.699	3.4	314 ± 8
Matrimid^®^/MIM-TFSI	3.42 × 10^−8^	3.965	1.589	0.732	0.614	2.9	329 ± 12
Matrimid^®^/EIM-TFSI	4.14 × 10^−8^	3.566	1.672	0.777	0.545	2.6	335 ± 8
Matrimid^®^/PIM-TFSI	4.98 × 10^−8^	2.478	1.783	0.801	0.467	3.5	357 ± 9
Matrimid^®^/BIM-TFSI	5.78 × 10^−8^	0.937	1.900	0.841	0.304	2.0	346 ± 7

**Table 6:** Fitted values of the ionic conductivity for the Matrimid^®^/PIL membranes according to the VFT equation (Equation (4)).

PIL/Parameter	*σ_0_*, S/cm	*T_0_*, K	*E_a_*, keV
MIM-TFSI	0.0074	216.8	32.91
	0.0034	269.3	7.56
EIM-TFSI	0.0066	194.1	28.81
PIM-TFSI	0.0083	119.6	56.69
BIM-TFSI	0.0098	119.6	56.69

## Supplementary Materials

The following are available online at https://www.mdpi.com/2077-0375/10/5/82/s1, Figure S1: TGA (a) and DTG (b) curves for synthesized PILs.
