# Supplementary material for: Polyimide/Ionic Liquid Composite Membranes for Middle and High Temperature Fuel Cell Application: Water Sorption Behavior and Proton Conductivity
Source: Membranes (Basel). 2020 Apr 28;10(5):82. doi: 10.3390/membranes10050082 (PMC7281338; doi:10.3390/membranes10050082)
Supplement: Supplementary file 1 [file membranes-10-00082-s001.pdf]

Supporting Information

Polyimide/ionic liquid composite membranes for middle and high temperature fuel cell application: water sorption behavior and proton conductivity

K. Fatyeyeva [a]*, S. Rogalskyy [b], S. Makhno [c], O. Tarasyuk [b], J. A. Soto Puente [a], S. Marais [a]

[a] Normandie Univ, UNIROUEN, INSA Rouen, CNRS, Polymères, Biopolymères, Surfaces (PBS), 76000 Rouen, France

[b] Institute of Bioorganic Chemistry and Petrochemistry, National Academy of Science of Ukraine, 50, Kharkivske schose, 02160 Kyiv, Ukraine

[c] Chuiko Institute of Surface Chemistry, National Academy of Sciences of Ukraine, 17, General Naumov St., 03164 Kyiv, Ukraine

*Corresponding author: kateryna.fatyeyeva@univ-rouen.fr

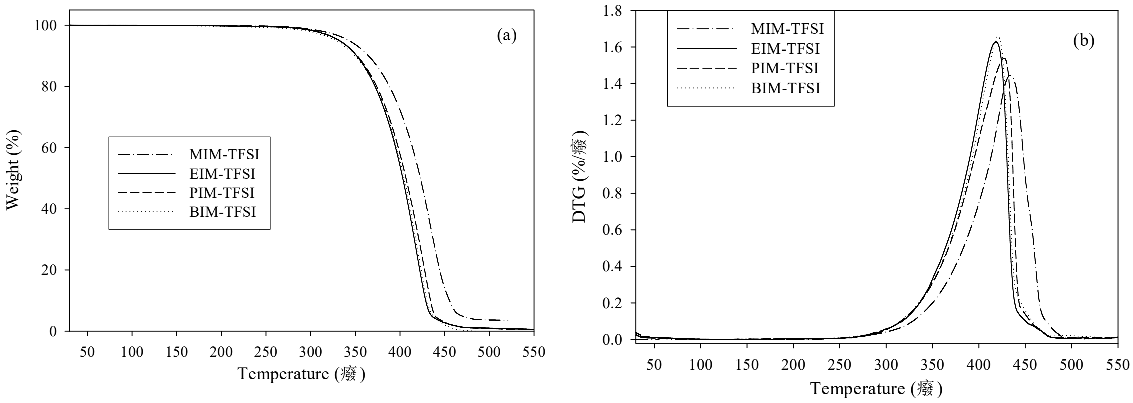

Figure S1. TGA (*a*) and DTG (*b*) curves for synthesized PILs.
